# Romiplostim for treatment of thrombocytopenia in dogs: A retrospective assessment and clinical outcomes

**DOI:** 10.1111/jvim.17131

**Published:** 2024-06-22

**Authors:** Min‐Ok Ryu, Jin‐Kyung Kim, Ju‐Hyun An, Kyoung‐Won Seo, Ye‐In Oh, Hwa‐Young Youn

**Affiliations:** ^1^ Laboratory of Internal Medicine, Department of Veterinary Clinical Science, College of Veterinary Science and Research Institute for Veterinary Science Seoul National University Seoul 08826 Republic of Korea; ^2^ Haemaru Companion Animal Medical Foundation Seongnam Republic of Korea; ^3^ Department of Veterinary Emergency and Critical Care Medicine and Institute of Veterinary Science, College of Veterinary Medicine Kangwon National University Chuncheon‐si Republic of Korea; ^4^ Department of Veterinary Internal Medicine, College of Veterinary Medicine & Institute for Veterinary Biomedical Science Kyungpook National University, Daehakro 80 Daegu Republic of Korea

**Keywords:** canine, immune‐mediated, pancytopenia, thrombopoietin

## Abstract

**Background:**

Romiplostim, a thrombopoietin analog, is commonly used to treat immune‐mediated thrombocytopenia (ITP) in humans, but its use in dogs remains limited.

**Objectives:**

Evaluate the effects and adverse events of romiplostim administration in dogs with thrombocytopenia caused by various underlying diseases.

**Animals:**

Forty‐two client‐owned dogs with naturally occurring thrombocytopenia at 2 referral animal hospitals.

**Methods:**

Retrospective, multi‐institutional analysis to evaluate the outcomes of romiplostim treatment in dogs.

**Results:**

Among the dogs treated with romiplostim, 27 experienced an increase in platelet count and 26 maintained a platelet count within the reference range. Platelet count improvement was observed in various conditions: primary ITP (90%, n = 18/20), pancytopenia of unknown etiology (42.9%, n = 3/7), chemotherapy‐induced thrombocytopenia (50%, n = 3/6), babesiosis (100%, n = 1/1), radiotherapy‐induced thrombocytopenia (0%, n = 0/1), and disseminated intravascular coagulopathy (33.3%, n = 2/6). The median time for platelet recovery (>50 000/μL) after romiplostim administration was 4 days, and the median time for platelet count normalization was 7 days. Median hospitalization time for the improvement group (I) was 5 days. The survival‐to‐discharge rates were 85%, 40%, and 28.6% for dogs with primary ITP, secondary thrombocytopenia, and thrombocytopenia of unknown etiology, respectively.

**Conclusions and Clinical Importance:**

Romiplostim is a well‐tolerated and promising treatment for primary ITP in dogs, suggesting its potential as a valuable therapeutic option for dogs with thrombocytopenia caused by various underlying conditions. These findings emphasize the need for further research to optimize romiplostim dosing and understand its role in treating secondary thrombocytopenia and pancytopenia of unknown etiology.

**Abbreviations**
AEs: adverse events
APTT: activated partial thromboplastin time
CCNU: chloroethylnitrosourea
DIC: disseminated intravascular coagulopathy
hIVIG: human intravenous immunoglobulin
I: improvement
ITP: immune‐mediated thrombocytopenia
MMF: mycophenolate mofetil
MODS: multi organ dysfunction syndrome
NI: non‐improvement
PCR: polymerase chain reaction
PDS: prednisolone
PT: prothrombin time
SIRS: systemic inflammation response syndrome
TCC: transitional cell carcinoma
TP: thrombocytopenia
VLP: vincristine loading platelets

## 1 INTRODUCTION

Thrombocytopenia is a commonly encountered hematologic disorder in veterinary medicine.^1^ Platelets play a crucial role in hemostasis, and thrombocytopenia can result in spontaneous bleeding and compromised clot formation.^2^ Thrombocytopenia in dogs can have various underlying causes, including immune‐mediated thrombocytopenia (ITP), infectious diseases, drug‐induced reactions, neoplasia, and systemic inflammatory conditions.^1^ Given the diverse causes, a comprehensive differential diagnosis is crucial to identify the underlying cause and guide appropriate treatment. Treatment strategies for thrombocytopenia depend on the underlying cause and may involve immunosuppressive therapy, transfusion, and supportive care.

In human medicine, romiplostim, a fusion protein analog of thrombopoietin, is an effective therapeutic option for ITP.^3^, ^4^, ^5^ Romiplostim acts by stimulating platelet production and maturation, resulting in increased platelet count and decreased risk of bleeding episodes.^6^ Although extensively studied in humans, literature regarding romiplostim use in dogs with thrombocytopenia is limited. In veterinary medicine, only 1 case series of romiplostim use for ITP in 5 dogs^7^ and a single case report on romiplostim administration for refractory amegakaryocytic thrombocytopenia exist.^8^

We aimed to retrospectively evaluate the effectiveness of romiplostim in correcting naturally occurring thrombocytopenia in dogs.

## 2 MATERIALS AND METHODS

### 2.1 Study design and patient selection

We retrospectively reviewed the medical records of dogs that received romiplostim between April 2021 and March 2023 at Seoul National University Veterinary Medical Teaching Hospital and Haemaru Referral Animal Hospital. The hospital board of the Seoul National University Veterinary Medical Teaching Hospital approved the study design and use of the medical records of the dogs. The owners of the dogs provided informed consent for participation and data publication. The Animal Ethics Committee of the Seoul National University Veterinary Medical Teaching Hospital approved the study. Dogs were included if they received romiplostim for treatment of thrombocytopenia.

### 2.2 Diagnostic evaluations and classification of thrombocytopenia

All dogs with confirmed thrombocytopenia were tested to determine the cause of thrombocytopenia and were categorized into 3 groups based on cause: primary thrombocytopenia, secondary thrombocytopenia, and unknown etiology of thrombocytopenia. Comprehensive diagnostic evaluations were performed on all included dogs to identify the underlying cause of thrombocytopenia. These included physical examination, CBC, reticulocyte count, serum biochemistry profile, C‐reactive protein (CRP), urinalysis, blood smear evaluation, SNAP 4Dx kit testing (IDEXX Laboratories, Westbrook, Maine, USA), vector‐borne disease PCR (*Anaplasma platys*, *Babesia gibsoni*, *Babesia* spp., *Ehrlichia* spp., Canine Hemotropic Mycoplasma, *Dirofilaria immitis*, *Hepatozoon* spp., *Rickettsia* spp., *Bartonella* spp., *Borellia* spp., and severe fever with thrombocytopenia syndrome virus), thoracic and abdominal radiographs, and abdominal ultrasonography. In some cases, fecal occult blood tests were performed to rule out intestinal hemorrhage. In some cases, coagulation panels, such as d‐dimers and prothrombin time (PT) and activated partial thromboplastin time (aPTT), also were evaluated. Platelet clumping‐induced pseudo‐thrombocytopenia was ruled out by blood smear examination.

### 2.3 Diagnosis criteria for primary and secondary thrombocytopenia

Primary ITP was diagnosed after excluding all other causes including infectious diseases, tumor‐related causes, hepatic or splenic sequestration, and any hemorrhagic conditions. Infectious causes were screened using blood smear analysis, 4Dx kit testing, and vector‐borne disease PCR. Tumor‐related causes were ruled out by physical examination, thoracic and abdominal radiography, and abdominal ultrasonography. Organ sequestration was assessed by abdominal radiography and ultrasonography. Any hemorrhagic conditions were evaluated by history taking, physical examination, thoracic and abdominal radiography, abdominal ultrasonography, urinalysis, and fecal occult blood testing.

Dogs that did not meet the diagnostic criteria for primary ITP were identified as having secondary thrombocytopenia or thrombocytopenia of unknown etiology. Dogs were categorized as having secondary thrombocytopenia when a specific cause was identified. Especially in cases where the dog had an underlying disease that could induce disseminated intravascular coagulopathy (DIC), and when considering the comprehensive results of packed cell volume (PCV), platelet, PT, aPTT, d‐dimer, and thromboelastography, if a subclinical or acute form of DIC was suspected, it was classified as DIC‐induced thrombocytopenia. Thrombocytopenia with leukopenia and anemia was categorized as unknown origin of pancytopenia in cases where owners declined advanced diagnostic procedures such as bone marrow aspiration for a definitive diagnosis.

### 2.4 Data collection from medical records

The following information was collected from each medical record: signalment, thrombocytopenia cause, concurrent diseases, romiplostim dosage and administration frequency, response to romiplostim, disease‐free interval, survival time, other medications prescribed to increase platelet count before romiplostim, adverse effects (AEs) of romiplostim, number of transfusions, and successful treatment for thrombocytopenia. The data collected before and during romiplostim treatment included CBC and serum biochemistry profiles.

### 2.5 Response evaluation and outcome measures

Dogs treated with romiplostim were categorized into improvement group (I) and non‐improvement group (NI). Improvement was defined as achieving a platelet count >50 000/μL or an increase in platelet count >150 000/μL if the baseline platelet count was >50 000/μL. The time to platelet normalization was defined as the difference between the date when the platelet count exceeded 150 000/μL and the date of drug injection. The platelet recovery was calculated based on the time when the absolute platelet count increased to >50 000/μL. The disease‐free interval was defined as the period from the date the platelet count returned to normal to the time of thrombocytopenia recurrence. Survival time was calculated from the time of diagnosis of thrombocytopenia until death, and, for patients that did not die, until last follow‐up.

### 2.6 Statistical analysis

Statistical analyses were performed using SPSS for Windows (version 26; IBM Corp., Armonk, New York). A Chi‐squared test of independence was performed to examine the relationship between survival rate and response to romiplostim. All data were presented as medians and ranges. Statistical significance was set at *P* < .05.

## 3 RESULTS

In our retrospective study, we analyzed the medical records of dogs prescribed romiplostim for thrombocytopenia between April 2021 and March 2023. Forty‐two dogs were identified. These cases were categorized based on the cause of thrombocytopenia: 20 dogs had primary thrombocytopenia, 15 had secondary thrombocytopenia, and 7 had thrombocytopenia of unknown origin. Table 1 summarizes the signalment data for the dogs in the study. The population comprised 20 castrated males, 17 spayed females, 4 intact females, and 1 intact male. The median age of the total population was 10 (range, 1‐14) years, and the main breeds were Maltese (n = 12), Miniature Poodle (n = 9), Miniature Schnauzer (n = 4), and Pomeranian (n = 3).

**TABLE 1:** Signalment data of dogs in this study.

Category of TP	n	Breeds (n)	Age (median, range)	Sex (n)	Detailed cause of TP (n)	Previous medication for TP (n)
Primary	20	Maltese (8), Miniature Poodle (3), Bichon Frise (2), Miniature Schnauzer (2), Pomeranian (2), Mixed (2), Spitz (1)	9.5 (1‐14)	FS (10), MC (7), IF (2), IM (1)	Idiopathic immune‐mediated thrombocytopenia (20)	PDS (18), vincristine (9), cyclosporin (4), MMF (9), leflunomide (8), hIVIG (13), melatonin (9), azathioprine (3), VLP (1)
Secondary	15	Miniature Poodle (5), Maltese (2), Miniature Schnauzer (2), American Bully (1), Chihuahua (1), Cocker Spaniel (1), Jack Russel Terrier (1), Standard Poodle (1), Yorkshire Terrier (1)	10 (6‐14)	MC (9), FS (5), IF (1)	Chemotherapy induced (6), DIC (6), Radiotherapy induced (1), Babesiosis (1), Toxication (1)	PDS (7), vincristine (3), leflunomide (2)
Unknown	7	Maltese (2), Miniature Poodle (1), Mixed (1), Papillon (1), Pomeranian (1), Spitz (1)	8.5 (6‐14)	MC (4), FS (2), IF (1)	Unknown pancytopenia (7)	PDS (5), vincristine (2), MMF (2), leflunomide (3), hIVIG (1)
Total	42					

Abbreviations: DIC, disseminated intravascular coagulopathy; FS, female spayed; GI, gastrointestinal; hIVIG, human intravenous immunoglobulin; IF, intact female; IM, intact male; ITP, immune‐mediated thrombocytopenia; MC, male castrated; MMF, mycophenolate mofetil; TP, thrombocytopenia; VLP, vincristine‐loaded platelets.

The differential diagnoses of thrombocytopenia included idiopathic primary ITP (n = 20), chemotherapy‐induced thrombocytopenia (n = 6), DIC (n = 6), radiotherapy‐induced thrombocytopenia (n = 1), Babesia‐induced thrombocytopenia (n = 1), toxication‐induced thrombocytopenia (n = 1), and pancytopenia of unknown etiology (n = 7).

Ten of the 20 dogs with primary ITP had comorbidities, including chronic gastritis, chronic enteritis, chronic pancreatitis, chronic hepatitis, seizures, urolithiasis, tracheal collapse, myxomatous mitral valve dysplasia (MMVD), and uterine fluid accumulation without inflammation, which were likely not associated with thrombocytopenia. One dog had MMVD that had been diagnosed as the American College of Veterinary Internal Medicine (ACVIM) B1 stage by echocardiography after heart murmurs were detected during a health examination a year before. The dog had undergone echocardiography every 3 months, confirming that MMVD remained ACVIM stage B1 without any enlargement of the left atrium. One dog with uterine fluid accumulation presented with petechial hemorrhage upon admission and had a platelet count of 0. Abdominal ultrasonography disclosed enlargement at the uterine horn and the presence of echogenic fluid. The dog's body temperature and CRP concentration were within normal ranges. Considering the dog had not been spayed but had been doing well over the past year, the likelihood of mucometra or hydrometra was considered high. Given the normalization of platelet count after treatment, the dog was classified as having primary ITP.

Dogs with secondary thrombocytopenia had concurrent diseases. Among them, 6 dogs exhibited thrombocytopenia associated with DIC. Three of these 6 dogs had systemic inflammatory response syndrome (SIRS) or sepsis associated with acute pancreatitis and peritonitis, pneumonia, or unknown cause. Of the remaining 3 dogs, 1 had acute pancreatitis and hepatic insufficiency, another had diabetic ketoacidosis, hepatic insufficiency, and hypothyroidism, and the other had acute kidney injury. Among the 6 dogs with chemotherapy‐induced thrombocytopenia, 1 had a malignant plasma cell tumor, 2 had multicentric lymphoma, and 3 had transitional cell carcinoma (TCC). The underlying disorder was multicentric lymphoma in 1 dog with radiotherapy‐induced thrombocytopenia. One dog with lymphoma received lomustine (chloroethylnitrosourea) and developed thrombocytopenia at a cumulative dose of 176 mg/m^2^, and romiplostim was administered after a cumulative dose of 213 mg/m^2^. Another dog with lymphoma developed thrombocytopenia 1 week after receiving vincristine as part of the cyclophosphamide‐doxorubicin‐vincristine‐prednisone (CHOP) protocol, leading to the administration of romiplostim. A dog with TCC was treated with romiplostim 16 days after receiving carboplatin because of thrombocytopenia. Another dog with TCC experienced thrombocytopenia 1 week after receiving a dose of mitoxantrone at 5 mg/m^2^, and romiplostim was administered 11 days after mitoxantrone treatment. Additionally, a dog with TCC developed pancytopenia after a single dose of mitoxantrone at 5 mg/m^2^, and romiplostim was administered 2 months after the onset of pancytopenia. In the dog with a malignant plasma cell tumor, daily administration of melphalan at a dosage of 0.05 mg/kg resulted in thrombocytopenia 200 days after the commencement of chemotherapy, at a cumulative dose of 10 mg/kg. Romiplostim treatment was initiated 28 days after the onset of thrombocytopenia.

Drugs used to treat thrombocytopenia before romiplostim included prednisolone (PDS), cyclosporine, leflunomide, mycophenolate mofetil (MMF), human intravenous immunoglobulin (hIVIG), melatonin, and vincristin‐loaded platelets (Table 1).

Blood transfusions were administered to 28 dogs (median, 2.5 times; range, 1‐13) before romiplostim treatment. For dogs with severe anemia caused by thrombocytopenia, with a PCV <20% necessitating transfusion, 20 mL/kg of whole blood or 10 mL/kg of packed red blood cells were given.

Table 2 summarizes the romiplostim administration protocols and reactions. Romiplostim was administered SC at a dosage of 5‐10 μg/kg, except for 1 dog that received 3 μg/kg. Twenty‐nine dogs received a single dose of romiplostim, whereas 13 received multiple doses (median, 1 dose; range, 1‐4 doses). Among the dogs that received multiple doses of romiplostim, 9 received the same dose, and 4 received an increased dose over time. The median platelet count before romiplostim treatment was 5000/μL (range, 0‐73 000), with 27 of 42 dogs (64.3%) having improved platelet counts after romiplostim administration. Among the I group, platelet counts in 1 dog, with pancytopenia of unknown etiology, improved after the first and second administrations, but did not improve after the third and fourth administrations. Additionally, 1 of the I group dogs, also with pancytopenia of unknown etiology, died from septic shock before the platelet count normalized (>150 000/μL). Consequently, 26 dogs achieved and maintained a normal platelet count. Platelet recovery (>50 000/ μL) was detected on a median of day 4 (range, 1‐7), and median platelet normalization was achieved on day 7 (range, 2‐12). Median duration of hospitalization for the I group was 5 (range, 2‐21) days. Follow‐up monitoring was conducted daily for hospitalized dogs, and every 2 to 7 days for dogs where hospitalization was refused by the owner or outpatient treatment was chosen because of an increase in platelet count and stabilization of vital signs.

**TABLE 2:** Platelet improvement after romiplostim administration in dogs with thrombocytopenia.

Category of TP	n	Response rate (n)		Dosage^a^ (μg/kg)	Administration time (day)	Pre‐treatment platelet (×10^3^/μL)	Platelet recovery (day)	Platelet normalization (day)
Primary	20	I	90% (18)	5 (5‐10)	1 (1‐3)	0 (0‐73)	4 (1‐7)	6.5 (2‐10)
		NI	10% (2)	5	1.5 (1‐2)	4.5 (0‐9)	‐	‐
Secondary	15	I	40% (6)^b^	5 (5‐10)	1 (1‐4)	14 (5‐67)	3.5 (1‐7)	7 (6‐12)
		NI	60% (9)	5 (3‐10)	1 (1‐4)	6 (0‐28)	‐	‐
Unknown	7	I	42.9% (3)^c^	5	1 (1‐4)	35 (20‐62)	5 (4‐6)	7
		NI	57.1% (4)	5 (5‐10)	1.5 (1‐4)	1 (0‐11)	‐	‐
Total	42	I	64.3% (27)	5 (5‐10)	1 (1‐4)	5 (0‐73)	4 (1‐7)	7 (2‐12)
		NI	35.7% (15)	5 (3‐10)	1 (1‐4)	3.4 (0‐28)	‐	‐

*Note*: The data are shown as median and range.

Abbreviations: I, improvement group; NI, non‐improvement group; TP, thrombocytopenia.

^a^ Based on the final administration to the dog when the dose was increased.

^b^ One dog responded to the first and second administrations of romiplostim but was nonresponsive to the third and fourth administrations.

^c^ One dog initially responded to romiplostim but became nonresponsive after the second administration.

Follow‐up monitoring included assessments of appetite, bowel and urination patterns, weight, blood pressure, heart rate, respiratory rate, body temperature, and tests such as CBC, blood smear, liver panel (alanine aminotransferase, aspartate aminotransferase, alkaline phosphatase, and gamma‐glutamyltransferase activity, and total bilirubin concentration), kidney panel (blood urea nitrogen, creatinine, calcium, and phosphorus concentrations), and electrolyte concentrations as standard procedures. Five dogs died after administration of romiplostim. Four of these 5 dogs were in the secondary thrombocytopenia group, and 1 dog belonged to the group with thrombocytopenia of unknown etiology. Given that the dogs' conditions were not stable even before the administration of romiplostim, it is difficult to definitively establish a causal relationship between the administration of romiplostim and their deaths. However, the possibility of AEs from romiplostim administration also should be considered. Aside from these 5 dogs, no other AEs were observed in clinical signs, physical examination findings, or blood tests after administration of romiplostim.

One dog developed thrombocytopenia associated with gastrointestinal bleeding and underwent hemodialysis for anuric acute kidney injury but died because of worsening pleural effusion and severe dyspnea. Three dogs suspected of having thrombocytopenia associated with DIC included 1 that, after ingesting toxic material and exhibiting polyuria and polydipsia, collapsed and was brought to the emergency room with hypovolemic hypernatremia and severe thrombocytopenia. This dog exhibited continuous seizures and worsening hypotension but died after the owner elected to discontinue treatment. Another dog with SIRS and severe hypernatremia was euthanized the next day because of a poor prognosis. A third dog died from worsening multiple organ dysfunction syndrome caused by sepsis. Additionally, 1 dog classified as having thrombocytopenia of unknown origin died from hypovolemic shock and persistent bloody feces.

In the dogs with primary ITP (n = 20), the platelet improvement rate after romiplostim treatment was 90% (n = 18). Median platelet recovery and median platelet normalization were detected on days 4 (range, 1‐7) and 6.5 (range, 2‐10), respectively. Median platelet counts before romiplostim treatment in the I and NI groups were 0/μL (range, 0‐73 000) and 4500/μL (range, 0‐9000), respectively.

The platelet improvement rate after romiplostim was 40% (n = 6) in the dogs with secondary thrombocytopenia (n = 15). The median duration of platelet recovery was 3.5 days (range, 1‐7), and the median duration of platelet normalization was 7 days (range, 6‐12). The median pre‐treatment platelet counts for the I and NI groups were 14 000/μL (range, 5000‐67 000) and 6000/μL (range, 0‐28 000), respectively.

The outcome after treatment in cases of secondary thrombocytopenia, where specific underlying causes of thrombocytopenia were identified, differed depending on the cause. In the 6 dogs diagnosed with DIC, 2 experienced improvements in platelet counts after administration of romiplostim. Notably, 1 of these dogs improved after the first dose but did not show an increase in platelet count from the second to the fourth doses. The other 4 dogs did not show any improvement in platelet count after treatment. In the 2 DIC cases categorized in the I group, the observed changes in thrombocytopenia might be more closely linked to the management of the underlying conditions, such as SIRS and acute liver injury, along with interventions including fresh frozen plasma and dalteparin administration. Based on the timing of these interventions and findings from serial therapeutic monitoring, the changes more likely were attributable to the management of DIC itself, rather than the effect of treatment with romiplostim.

In dogs that experienced bone marrow suppression associated with chemotherapy, overall assessment showed that thrombocytopenia improved in 3 of 6 dogs after administration of romiplostim. However, the 3 dogs in which thrombocytopenia improved (1 dog with TCC treated with mitoxantrone, 1 dog with TCC treated with carboplatin, and 1 dog with multicentric lymphoma treated with vincristine) were determined to have thrombocytopenia as an AE at the nadir period after chemotherapy administration. Although thrombocytopenia improved after romiplostim administration, this outcome could have been expected even without romiplostim treatment, making it difficult to attribute the improvement to romiplostim. In the 3 dogs in which thrombocytopenia did not improve, the dogs continued to experience thrombocytopenia for a longer period than the typical rebound phase after chemotherapy, suggesting the occurrence of bone marrow disorders induced by lomustine, melphalan, and mitoxantrone, respectively. In these instances, romiplostim appeared to be ineffective.

In secondary immune thrombocytopenia associated with babesiosis infection, the only dog in this category experienced improved platelet counts after romiplostim, but concurrent administration of antibiotics possibly contributed to the observed effects. The beneficial outcomes observed in this dog were possibly attributable to the combined therapeutic effects of the antibiotics and romiplostim. Lastly, the dog with radiotherapy‐induced thrombocytopenia improved after receiving romiplostim.

The survival‐to‐discharge rates were as follows: 59.5% (n = 25/42) for all dogs, 85% (n = 17/20) for those with primary ITP, 40% (n = 6/15) for those with secondary thrombocytopenia, and 28.6% (n = 2/7) for those with thrombocytopenia of unknown etiology. The survival‐to‐discharge rate was calculated based on the dog's status at discharge, including dogs that dropped out of treatment owing to the owner decisions or those that were transferred to a primary hospital (surviving dogs). The survival rate was 85.2% (n = 23) in the I group (n = 27), whereas that in the NI group (n = 15) was 0% (n = 0). The difference in survival rates between the I and NI groups was significant (*P* < .001).

## 4 DISCUSSION

The platelet improvement rates observed in our study provide valuable insight into the effectiveness of romiplostim administration in dogs with thrombocytopenia. We found a promising overall platelet improvement rate of 64.3% in the included dogs, indicating the potential of romiplostim as a therapeutic option for managing thrombocytopenia in dogs. The primary thrombocytopenia group experienced the highest improvement rate (90%), followed by the thrombocytopenia of unknown etiology group (42.9%) and the secondary thrombocytopenia group (40%).

We evaluated the combined use of romiplostim with other immunosuppressants for the treatment of primary ITP in dogs. The median time for platelet recovery (>50 000/μL) was 4 days when romiplostim was part of the treatment regimen. In comparison, previous research on the efficacy of various immunosuppressants for primary ITP in dogs has shown variable results. For dogs treated with hIVIG and corticosteroids, the median time for platelet recovery (>40 000‐50 000/μL) ranged from 2.5 to 5 days.^9^, ^10^, ^11^ Similarly, the combination of vincristine and corticosteroids resulted in a median platelet recovery time of 2.5 to 4 days.^9^, ^11^ For treatments involving azathioprine and corticosteroids, platelet recovery was achieved in 6 days,^11^ whereas corticosteroids alone produced recovery after 7.5 days.^10^ Given that our study was not a case‐control study, we cannot definitively state that the use of romiplostim leads to better outcomes compared to not using it. However, our findings suggest that platelet recovery may occur earlier when romiplostim is used with other immunosuppressants for treating primary ITP in dogs.

In our study, administering romiplostim along with other immunosuppressants to dogs with primary ITP resulted in a median platelet normalization (>150 000/μL) time of 6.5 days. This result compares favorably to durations reported in previous studies involving various immunosuppressive treatments. For example, dogs treated with MMF and corticosteroids achieved platelet normalization (>148 000‐160 000/μL) within 10 days.^12^ Dogs on a regimen of cyclosporine and corticosteroids achieved normalization within 9 days.^12^ Those receiving azathioprine and corticosteroids required 15 days for median normalization, whereas the combination of vincristine and corticosteroids resulted in normalization in 10 days.^11^ Dogs treated with hIVIG and corticosteroids experienced normalization within 8 to 12 days, and those treated exclusively with prednisolone required 13 days for normalization.^10^, ^11^ Thus, the use of romiplostim in combination with other immunosuppressants may shorten the time to platelet normalization in dogs with primary ITP.

In our study, the survival‐to‐discharge rate was 85% in dogs with primary ITP (n = 20). Others have reported survival‐to‐discharge rates of 70%‐83% in dogs with primary ITP treated with corticosteroids and hIVIG,^9^, ^11^ 70% in dogs with primary ITP treated with corticosteroids and azathioprine,^11^ and 90% in dogs with primary ITP treated with corticosteroids and vincristine.^11^ Several studies that compared the effectiveness of different drugs found no significant differences in survival‐to‐discharge rates based on the drug used.^9^, ^10^, ^11^, ^12^ The survival‐to‐discharge rates of dogs with primary ITP treated with romiplostim were similar to those treated with other regimens, as other studies have shown.

In contrast, dogs with secondary thrombocytopenia or pancytopenia of unknown etiology had relatively lower survival‐to‐discharge and response rates compared to those with primary ITP. For the 3 patients classified as I group with chemotherapy‐induced thrombocytopenia, it is likely that platelet counts would have rebounded even without romiplostim, based on its administration timing at the nadir after chemotherapy. Therefore, the actual response rate may be lower than what was recorded. Additionally, in the case of a dog with babesiosis, platelet normalization may have occurred primarily because of antibiotic treatment rather than romiplostim, emphasizing the importance of treating the primary cause of thrombocytopenia in dogs with secondary thrombocytopenia. The 3 dogs with chemotherapy‐induced thrombocytopenia that did not respond to romiplostim exhibited prolonged thrombocytopenia beyond the typical rebound period after conventional chemotherapy treatment, suggesting the possibility of a bone marrow disorder. Similarly, for dogs with pancytopenia of unknown etiology, accurate diagnoses were not achieved because of the lack of bone marrow aspiration cytology or biopsy results, but bone marrow abnormalities were suspected. Romiplostim may not be effective for thrombocytopenia caused by bone marrow disorders. However, given the very small number of cases in the groups with secondary thrombocytopenia and pancytopenia of unknown etiology, it is not possible to generalize these findings.

Additional studies with larger numbers of dogs and a more balanced distribution of causes of secondary thrombocytopenia are warranted to evaluate the effectiveness of romiplostim in these situations. More extensive analysis of romiplostim treatment outcomes in dogs with secondary thrombocytopenia may provide insight into its potential benefits and limitations for different underlying causes of thrombocytopenia. Considering the diverse causes of secondary thrombocytopenia, future research should focus on establishing standardized diagnostic criteria and treatment protocols tailored to each cause, enabling more comprehensive investigation of treatment outcomes.

In our retrospective study of romiplostim use in dogs with thrombocytopenia, we assessed the occurrence of AEs. Adverse events reported in human patients include headache, fatigue, ecchymosis, epistaxis, petechiae, arthralgia, dizziness, insomnia, and myalgia.^3^, ^5^, ^6^, ^13^ Because our study was conducted on dogs, identification of AEs such as headaches, fatigue, and dizziness was impossible. However, no cases of ecchymosis, epistaxis, or other clinical signs were observed after romiplostim treatment in our study population, indicating that romiplostim treatment was generally well tolerated by the dogs. These findings suggest a favorable safety profile for romiplostim in dogs. Given the AEs associated with romiplostim use in humans, dogs receiving romiplostim should be monitored for potential AEs. Additionally, deterioration and death associated with underlying diseases occurred after romiplostim use in 4 dogs in the secondary thrombocytopenia group (3 with DIC and 1 with intoxication) and in 1 dog with pancytopenia of unknown etiology, including 2 dogs in which euthanasia was elected because of the owners' decision to discontinue treatment. Although a direct causal relationship was not definitively established, the possibility of AEs related to romiplostim use should be considered.

Our retrospective study was limited by the lack of a standardized protocol for romiplostim administration. Variations in dosage and frequency among the cases might have contributed to variability in treatment outcomes. Additionally, the concurrent use of other medications with romiplostim prevents identification of the specific effects of romiplostim alone. Another important limitation is that our study, being retrospective and consisting of clinical patients rather than being part of a prospective clinical trial, lacked a control group. Absence of a control group makes it impossible to directly compare the outcomes of dogs treated with romiplostim in combination with other immunosuppressants to those not receiving romiplostim, thereby limiting definitive conclusions about its efficacy. Future prospective studies, featuring larger sample sizes and appropriate control groups, are necessary to clarify the potential AEs and safety profile of romiplostim in dogs. Furthermore, long‐term follow‐up studies would offer valuable information regarding the sustainability of the treatment response and the recurrence rates of thrombocytopenia.

In conclusion, our retrospective study suggests that romiplostim, in combination with other immunosuppressants, is well‐tolerated and shows promise in managing primary ITP in dogs. However, outcomes related to efficacy and survival in cases of secondary thrombocytopenia and pancytopenia of unknown etiology were comparatively modest, emphasizing the importance of further research to explore romiplostim's potential value for various causes of thrombocytopenia. These findings emphasize romiplostim's potential as a therapeutic option as well as the need for prospective studies to more comprehensively assess its efficacy and safety profile in a broader range of thrombocytopenic conditions in dogs.

## CONFLICT OF INTEREST DECLARATION

Authors declare no conflict of interest.

## OFF‐LABEL ANTIMICROBIAL DECLARATION

Authors declare no off‐label use of antimicrobials.

## INSTITUTIONAL ANIMAL CARE AND USE COMMITTEE (IACUC) OR OTHER APPROVAL DECLARATION

Authors declare no IACUC or other approval was needed.

## HUMAN ETHICS APPROVAL DECLARATION

Authors declare human ethics approval was not needed for this study.
